# Mapping Inequalities in the Physical, Built and Social Environment in Population-Based Studies of Brain Health

**DOI:** 10.3389/fnimg.2022.884191

**Published:** 2022-04-26

**Authors:** Tomáš Paus, Jeff Brook, Dany Doiron

**Affiliations:** ^1^Departments of Psychiatry and Neuroscience, Faculty of Medicine and Centre Hospitalier Universitaire Sainte-Justine, University of Montreal, Montreal, QC, Canada; ^2^Departments of Psychology and Psychiatry, University of Toronto, Toronto, ON, Canada; ^3^Dalla Lana School of Public Health, University of Toronto, Toronto, ON, Canada; ^4^Respiratory Epidemiology and Clinical Research Unit, Research Institute of the McGill University Health Centre, Montréal, QC, Canada

**Keywords:** physical environment, built environment, social environment, brain development, epidemiology, MRI, mental health, inequality

## Abstract

This mini-tutorial describes how combining aggregate-level data about the physical, built and social environment can facilitate our understanding of factors shaping the human brain and, in turn, brain health. It provides entry-level information about methods and approaches one can use to uncover how inequalities in the local environment lead to health inequalities in general, and those in brain health in particular. This background knowledge should be helpful to those who are interested in using neuroimaging to investigate how environmental factors shape inter-individual variations in the human brain.

## Introduction

An individual's brain development and aging – and brain health - are influenced by context. Outside the immediate family, many impactful contextual factors influencing our everyday lives act where we live and work. Area-level characteristics of physical, built and social environments shape our brains from conception onwards. But not all areas are the same: inequalities exist in multiple inter-twinned dimensions.

Social, economic and political conditions produce *health inequalities* within and across countries (Stuart and Soulsby, 2011; Scambler, 2012; Metzl and Hansen, 2018). For example, in high-income countries, individuals are more likely to experience poor mental health if growing up in households with low income (Bjorkenstam et al., 2017) or affluence (Rajmil et al., 2014; Elgar et al., 2015), living in areas with high deprivation (Kivimaki et al., 2020) or experiencing inequalities in income distribution (Mangalore et al., 2007). Certain communities are disadvantaged more than others (Waldron, 2018). This is especially true for Indigenous (Ogilvie et al., 2021) and racialized (Castro-Ramirez et al., 2021) communities, with a higher risk of mental-health problems and – at the same time – lower likelihood of receiving evidence-based treatment (Castro-Ramirez et al., 2021). At the area level, our physical, built and social environments combine to create ecosystems in which we live and work. Together, these ecosystems – and the structures and systems that produce them – contribute to what has been termed “social and structural determinants of health” (Vandenbroucke, 1990; Diderichsen et al., 2001).

As we have described elsewhere (Paus, 2016), there are countless permutations of physical, built and social environments that surround us in space and time. We both “receive” and “create” our environments (Kendler et al., 2003), thus co-determining what air we breathe, how many steps we take, how hot or cold we are, what and who we see, hear and interact with during our commutes. Together with our genes, these “external exposures” contribute to “internal” environments that exist in our body: on body surfaces (e.g., microbes on our skin and in the gut), in the lungs (e.g., particulate matter), circulating blood (e.g., toxins, micronutrients, inflammatory molecules) and the brain (e.g., stress- and reward-related neurotransmitters, cumulative engagement of specific neural circuits).

Assessing the “external” environment – rather than its biological markers in biospecimen – is challenging. Asking a series of questions using a standard survey is a common way of collecting information about the individual's physical, built and social environment. The PhenX Toolkit, for example, contains standardized protocols (including surveys) one can use to collect information about social determinants of health (www.phenxtoolkit.org/index.php). Although valuable, there are two main disadvantages of a survey-based approach: (1) participant's time (many hours required to cover multiple domains); and (2) self-reported nature of the collected information and, therefore, possible reporting biases. Furthermore, with the exception of longitudinal (birth) cohorts, questionnaire-based approaches provide only a snapshot of environments encountered by the individual at one (or a few) timepoints.

Here we describe an alternative approach, namely the use of aggregate-level (spatial) data, produced for multiple locations and time points, to characterize physical, built and social environments. We will then provide a brief overview about the linkage of such aggregate-level data with individual-level information about person's health in general, and brain health in particular.

## Geospatial Mapping of Area-Level Environments

Geospatial science and related tools enable spatial analysis and visualization of external environments in which we spend considerable amount of our lives (e.g., our residence, place of work, school, recreation or a commute path) and, in turn, evaluation of their impact on the individual's health. Datasets can be created at different levels of spatial granularity matching the goals of a given study and availability of relevant data. In Canada, for example, geographic units include six-digit postal codes, Canadian Census geographic units such as dissemination areas (DA; 400 to 700 persons) and census tracts (CT; 2,500 to 8,000 persons), or larger areas such as city districts. The spatial unit used to link geospatial datasets to health data varies; depending on the study and measures taken to protect confidentiality of study participants, this can be as precise as the exact street address or a postal code (half of a city block in dense urban area), or as coarse as a city district, a county, a province/state or a country. The temporal dimension depends on the type of data; it may range from data sampled monthly (e.g., air quality), annually (e.g., public transportation) or up to every 5 years (e.g., the Canadian Census).

**Figure 1:**
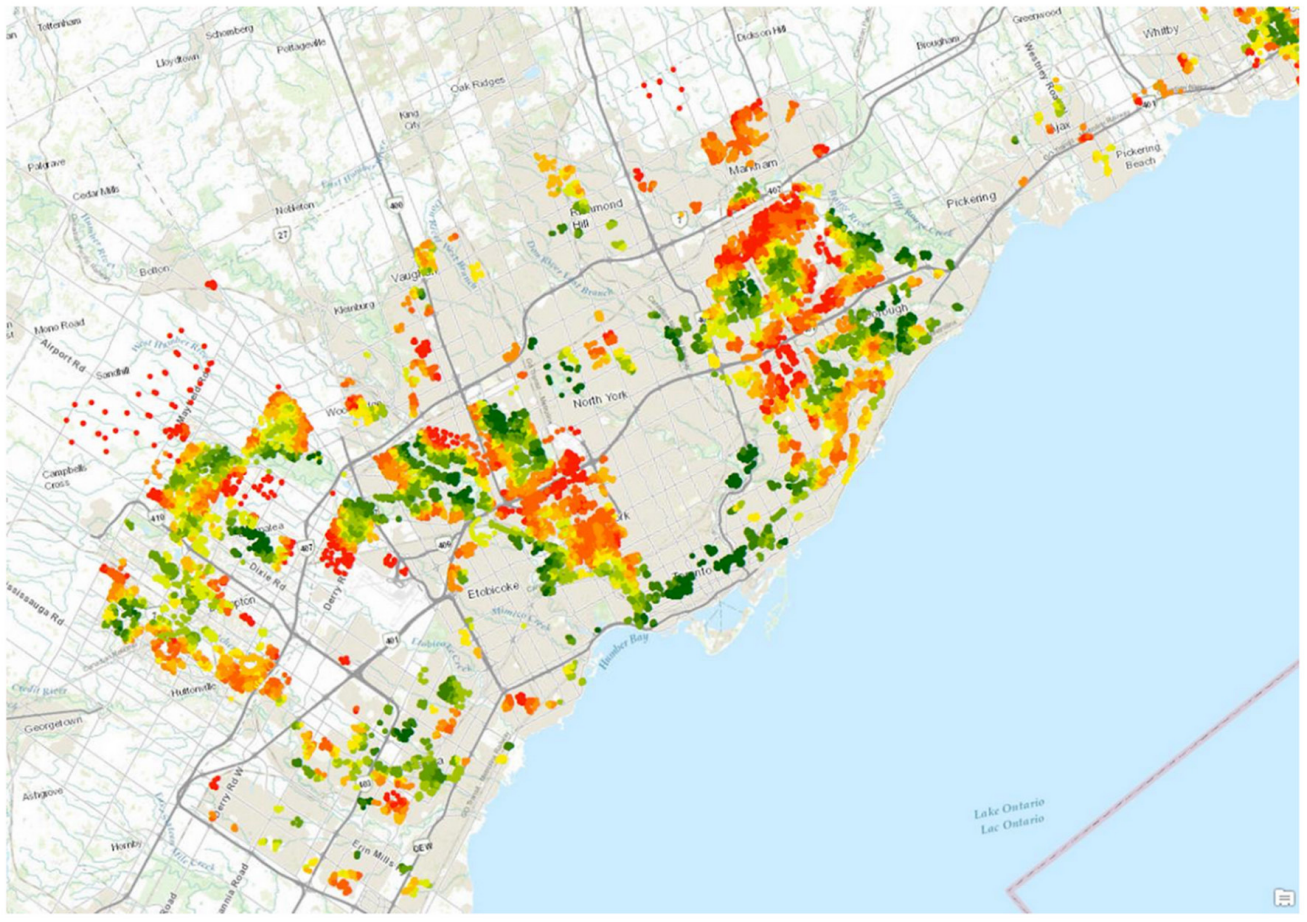
Material deprivation and access to parks and recreation, Greater Toronto Area. All colored areas represent postal codes characterized by high (top 20%) material deprivation [Source: Pampalon and colleagues (Pampalon et al., 2012)]. Green indicates postal codes in the highest 10% density of park and recreational amenity within 1 km, red indicates postal codes in the lowest 10% (Source: Open Street Map).

The spatio-temporal datasets can be created using existing tools and databases provided by large GIS-based (*Geographic Information Systems*) organizations and companies, such as ESRI (www.esri.com), DMTI Spatial (www.dmtispatial.com), Google Earth Engine (https://earthengine.google.com), as well as open sources (e.g., www.openstreetmap.org), government sources (e.g., Statistics Canada), and academic organizations. The raw (initial) datasets are typically curated by data specialists and GIS technicians. In Canada, we have acquired, curated and disseminated geospatially coded information about physical and built environment through the Canadian Urban Environmental Health Research Consortium, CANUE (www.canuedata.ca/metadata.php) (Brook et al., 2018). Metrics derived from different sources can be combined to ask, for example, questions about the relationship between socio-economic indicators (e.g., household income) and built environment (e.g., access to parks), and thereby used to assess inequity in the spatial distribution of environmental good or hazards (Doiron et al., 2020). Figure 1 illustrates inequality in the access to parks and recreation (derived from Open Street Map data) across areas with the same (high) level of material deprivation [derived from Canadian Census data (Pampalon et al., 2012)].

In addition to sourcing and creating data about physical and built environments from existing databases [see Table 1 in (Paus, 2016)], one can also derive relevant metrics from new data streams such as high-resolution satellite and street-level imagery combined with machine-learning techniques (Weichenthal et al., 2019). For example, *Google Street View* allows investigators to assess different features of built environment using panoramic street-level images taken mostly by camera-equipped cars, while recent satellite technology provides daily coverage of most inhabited areas on Earth at a resolution of only a few meters. These geo-coded images can be rated for various features, such as signs of physical disorder (e.g., litter, graffiti), physical decay (e.g., poor conditions of sidewalks), type of stores, traffic, or street walkability (Odgers et al., 2012; Less et al., 2015); there are some limitations of this approach, however (Curtis et al., 2013). In turn, these computer vision and machine learning algorithms can exploit these image data to generate indirect indices of social environment (e.g., psychosocial stress) and physical environment (e.g. air or noise pollution) in a manner similar to that used by others to derive metrics such as living environment, health and crime (Suel et al., 2019).

**Table 1:** Examples of measures, with the corresponding sources of raw geospatially coded data and examples of the new types of data to be derived.

**Physical and built environment**	**Social environment**
Air quality (NO2, O3, SO2, PM2.5)^1^	^b^Demographic^10^
Greenness (greenest pixel, tree canopy)^2^	^c^Households^11^
Night time light^3^	^d^Socioeconomic^12^
Noise^4^	Water quality concerns^13^
Public transportation^5^	Composting and recycling behavior^14^
Proximity to roads^6^	Involvement in outdoor activities^15^
Proximity to retail outlets and sales for alcohol, tobacco, cannabis and gambling^6^	Caregiving and care receiving^16^
Green roads^7^	Social identity^17^
Facility Index^8^	Giving, volunteering and participating^18^
^a^Cumulative Opportunities^9^	Victimization^19^
	Social-media and search-engine use by youth: Frequency and Time of day^20^
	Social-media and search-engine use by youth: Content^21^
	Built-environment predictors of psychosocial stress^22^
	Built-environment predictors of social cohesion^23^

1−3 *Landsat*;

4 *CANUE*;

5 *OpenStreetMap (OSM)*;

6 *DMTISpatial*;

7−9 *OSM and CANUE*;

10−12 *Census*;

13−15 *Household and the Environment Survey (Canada)*;

16−19 *The General Social Survey (Canada)*;

20, 21 *Newly derived measures from raw data streams (e.g., Twitter, Google search-engine)*;

22, 23 * Newly derived measures from raw data streams (satellite and street-view imagery)*;

a *Travel times (walking, public transport) to jobs, leisure, and shopping, as well as health, medical, and social services*;

b *Population (total and densities), Proportions (by age, sex, ethnicity, marital status, mobility/migration status, religion, mother tongue)*;

c *Household size, Total housing units, Proportion rented, Type of dwelling*;

d *Household income, Unemployment rate, Proportion below poverty line, Proportion (by age/sex) in labor force*.

As summarized in Table 1 (Social environment), there is a wealth of data that speak to basic – often self-reported - measures of socio-economic factors, such as education, employment, immigration, household spending habits or volunteering and giving, that are collected through governmental agencies (e.g., census) and national surveys. But one can also use data from digital streams, such as search-engine usage and Twitter, to generate new metrics of social environment relevant, for example, for attitudes vis-à-vis health and health interventions (e.g., vaccination), as well as social cohesion, social support and role models and, most recently, for the emerging issues related to environmental anxiety (Hickman et al., 2021; To et al., 2021; Soutar and Wand, 2022; Usher, 2022). The key advantage of this approach is its reliance on *behavior* rather than self-reports; as such, it is akin to ethology, a study of animal behavior by observing it in the “wild”. *Twitter* – for example - provides a rich source of information about the interests of its users; a short message (a tweet, up to 140 characters in length) sent by a user can be tagged by a hashtag identifying explicit topics of the message. One can retrieve all tweets posted or retweeted from a particular location during a 12-month period from Twitter's full firehose, using PowerTrack filtering language (), and: (1) map all Twitter users into geospatial units; (2) count the number of times each user posted or retweeted a tweet; (3) extract the tweet time-of-day; (4) determine the physical distance between each Twitter user and the geographic origin of a retweeted tweet; and (5) categorize tweets into various social themes, such as Economy, Politics, Health, Employment and Spending. The latter approach has been developed by Peng and colleagues in a study of interplay between real events and Twitter activity (Peng et al., 2017) whereby tweets are categorized using ~2,000 keywords related to the five social issues, followed by a machine-learning approach to filter out all irrelevant tweets (Peng et al., 2017). *Facebook* – an on-line social network – provides another window into social activities of its users. Various Facebook activities, such as wall postings, likes or status messages, can be used to classify users into different categories with regards to their interest, such as watching TV (Chunara et al., 2013), or their individual characteristics, such as the Big Five personality traits (Park et al., 2015). These can be, in turn, related to the geographic locations of Facebook users, thus creating additional layers of information about social features of a given geospatial unit. The above examples illustrate the power of *digital ethology*, an objective way of assessing social environment by measuring behavior through the individual's use of digital tools.

Once properly curated, all aggregate-level data (see Table 1 for examples) should be described using comprehensive metadata and indexed to different geographic units (postal codes, dissemination areas and other census geographies), as we have done previously (https://canuedata.ca/).

## Linkage With Individual-Level Data

Ultimately, one is interested in linking aggregate-level “exposures” described above to the individual-level “outcomes”. Here we provide two examples of how one can achieve this goal by using: (1) administrative health databases; and (2) data acquired in research cohorts.

### Administrative Data

Over the past 2 years, we have all seen the power of mapping administrative data related to COVID-19 (across countries, provinces/states or cities), and communicating these numbers to the public. In Canada, administrative health data – data that are captured in the course of providing services or running programs - are made available for research use by provincial governments and other agencies, often in close partnership with academic organizations (Lucyk et al., 2015). In all provinces, these data are longitudinal and population-based, covering all residents who have received health care and social services (e.g., education) - from birth onward. This creates comprehensive and important data for the population of interest, such as youth.

In the province of Ontario, for example, such administrative health data are curated and made available for research by ICES, “a not-for-profit research institute encompassing a community of research, data and clinical experts, and a secure and accessible array of Ontario's health-related data” (www.ices.on.ca). Behind a firewall, ICES provides access to de-identified databases containing, for example, the Ontario Mental Health Reporting System. Just in the City of Toronto, these data are available for about 270,000 adolescents & youth (12–22 years of age). In addition to health data, many of the provincial custodians of administrative data provide access to other linked datasets, such as education, workplace or justice data (https://www.popdata.bc.ca). When linking administrative data with geospatial datasets containing area-level characteristics of physical, built and social environment, one would typically use the residential six-digit postal codes (Canada) and relevant geographies (e.g. dissemination blocks) reported in the administrative data for each individual. Postal code-indexed geospatial datasets are linked in the secure environments controlled by the custodian of the individual-level health data. Ethical and legal guidance is necessary here to provide assurance to data stewards that this form of data linkage and access can be done in a privacy-preserving and transparent manner that respects all applicable legal, regulatory, and ethical requirements.

### Cohort Studies

One of the key advantages of administrative health data is their population-wide coverage. On the other hand, by definition, these data show only the tip of the “health iceberg”, namely individuals with health issues significant enough to enter the health-care system. This is where community-based cohort studies come in as a complementary source of information, with longitudinal birth cohorts being most valuable. For example, birth cohorts are well suited for investigating relationships between brain health (individual-level data) and context (aggregate-level characteristics of the environment) for several reasons: (1) many birth cohorts [e.g., ALSPAC (Boyd et al., 2013), Generation R (Tiemeier et al., 2012) and Northern Finland Birth Cohorts (Rantakallio, 1988)] ascertained their participants (pregnant women) in a relative small geographic area; (2) each cohort includes a relatively large sample size of individuals (~10,000); and (3) brain (e.g., mental) health of cohort members is assessed using a number of instruments, often on a continuous scale. The combination of the first two features guarantees that a reasonable number of participants lives in each geospatial unit, hence providing sufficient statistical power to investigate these relationships. The third feature (assessment) allows one to capture “subclinical” mental-health problems. Finally, additional deep-phenotyping of cohort members (e.g., cognitive assessment, neuroimaging, blood-based biomarkers [e.g., inflammation], genotyping and epigenotyping) provides rich information suitable for detailed modeling of exposure-outcome relationships and their mediators and moderators (Paus, 2013).

**Figure 2:**
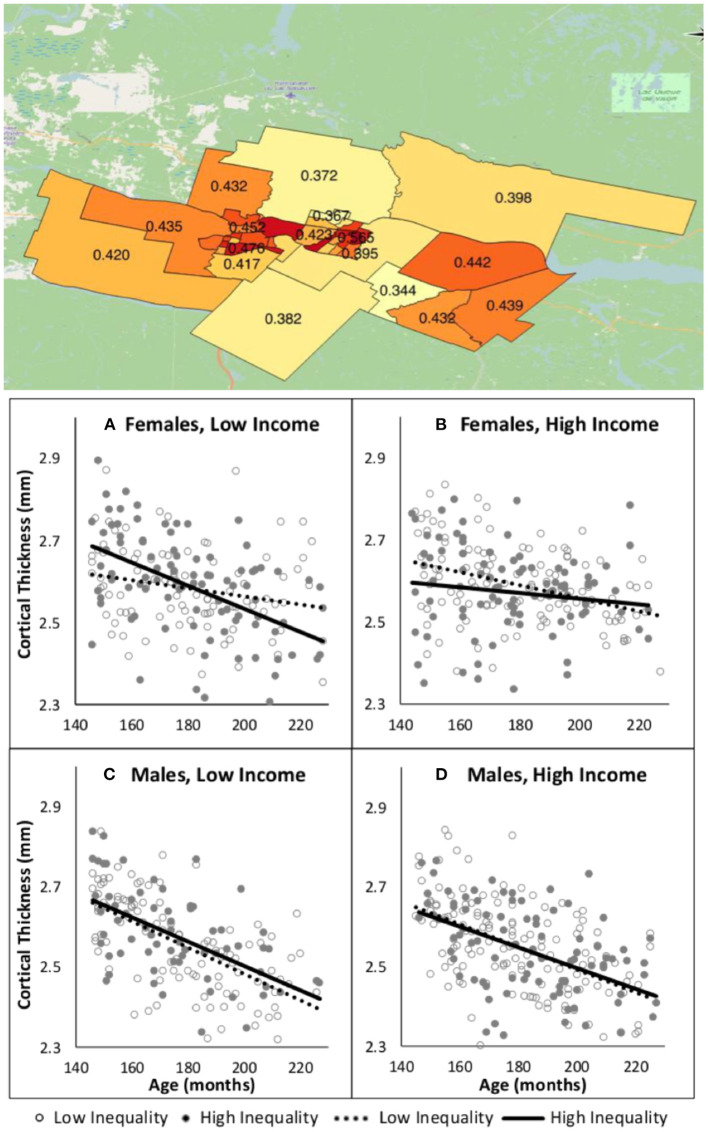
Income inequality and age-related changes in cortical thickness during adolescence [from Parker et al. (2017)]. **Top:** Values of Gini index (higher values indicate a larger gap between the low and high incomes) for 37 census tracts covering the region in which participants in this cohort study live. **Bottom:** Age-related changes in cortical thickness as a function of income inequality and household income in male and female adolescents. In each plot, household-income groups (low, high) are stratified by income inequality: high = solid circles and solid line, low = hollow circles and dashed line.

Using data from ongoing developmental cohorts, we and others have linked individual-level information with aggerate-level data to evaluate, for example, the relationship between income inequality and brain maturation during adolescence (Parker et al., 2017), the role of urbanicity in inter-individual variations in brain structure and function in youth (Xu et al., 2021), and the relationship between the risk of environmental exposure to lead and brain structure in childhood (Marshall et al., 2021). In the study on income inequality (Parker et al., 2017), for example, we linked information on income distribution (Gini index) in each census-based geospatial unit in a particular region with individual-level information (MRI-derived estimates of cortical thickness) for all participants living in this region. We found that female (but not male) adolescents living in census tracts with high income inequality showed a strong relationship between age and cortical thickness; this was only the case for females living in household with low income (Figure 2). We interpreted these findings as related to psychosocial stress associated with social comparisons whereby adolescents from low- and high-income households encounter each other frequently when living in areas with high income inequality (Parker et al., 2017). Several other studies and reviews have highlighted the potential of linking aggregate-level assessment of environment and individual-level (neuroimaging) data (Fan et al., 2021), and conceptualized the relationship between external environment and brain health (Tost et al., 2015; Berman et al., 2019).

We will close this section with a hypothetical example illustrating how one can use aggregate-level information about the physical, built and social environment to unpack the relationship between poverty and mental health. As pointed out by Diderichsen et al. (2001), social stratification – with poverty being but one example of social, economic and political inequalities – generates a vicious circle: (1) disadvantaged persons are more likely to be exposed to harmful or deprived physical (e.g., air pollution), built (e.g., access to food stores) and social (e.g., lack of social support, violence, mistreatment) environments and to population-level challenges (e.g., heat waves, Sars-CoV-2); (2) these exposures lead to an increased vulnerability to other exposures (e.g., bullying); and (3) exposures and vulnerabilities combined precipitate (mental) illness. The vicious circle is closed by the illness leading to further social stratification (e.g., lost educational and employment opportunities). Rich multi-domain datasets one can create using various data sources (see above) would enable testing a variety of possible pathways (and their combination) leading from social stratification to mental health; decomposition analysis is but one of the methods one can use to quantify contributions of various factors to the observed outcomes (Wagstaff et al., 2008).

## Conclusion

The above approach that combines aggregate-level assessments of “exposures” with individual-level information on “outcomes” reflects principles of “*Big Data*” and “*Open Science*”. We offer that no single study, however large, can achieve what is possible using this approach: (1) concurrent quantification of multiple standardized measures of physical, built and social environment; (2) large geospatial coverage (e.g., an entire country) implemented with a high geographic granularity; (3) extraction of the same measures at multiple timepoints, going back 20+ years; and (4) linkage of aggregate-level to individual-level data, the latter collected through administrative health databases and research cohorts. Although the availability of different types of data varies across space (geospatial granularity), time (frequency of sampling) and countries (monitoring tools, access), such limitations can be addressed – for example - by modeling (e.g., land-use regression to estimate air pollution) or through targeted surveys and other data collections. Overall, we suggest that this approach brings together two large communities of scholars and their trainees, namely health geographers with population neuroscientists, thus leveraging their respective knowledge and expertise to uncover pathways leading from our environment to brain health. It is clear that structural inequalities lead to health inequalities but the pathways mediating or moderating this relationship are largely unknown. A detailed, and concurrent, assessment of the physical, built and social environment on a large scale may allow one to approach the complexity of the dynamic relationship between “environment” and “external” and the human brain.

## Author Contributions

TP conceived this mini-review and wrote the first draft. JB and DD contributed to the first draft and reviewed critically the entire manuscript. All authors contributed to the article and approved the submitted version.

## Conflict of Interest

The authors declare that the research was conducted in the absence of any commercial or financial relationships that could be construed as a potential conflict of interest.

## Publisher's Note

All claims expressed in this article are solely those of the authors and do not necessarily represent those of their affiliated organizations, or those of the publisher, the editors and the reviewers. Any product that may be evaluated in this article, or claim that may be made by its manufacturer, is not guaranteed or endorsed by the publisher.
